# Viral genome wide association study identifies novel hepatitis C virus polymorphisms associated with sofosbuvir treatment failure

**DOI:** 10.1038/s41467-021-25649-6

**Published:** 2021-10-20

**Authors:** David A. Smith, Carlota Fernandez-Antunez, Andrea Magri, Rory Bowden, Nimisha Chaturvedi, Jacques Fellay, John McLauchlan, Graham R. Foster, William L. Irving, STOP-HCV Consortium, Jonathan Ball, Diana Brainard, Gary Burgess, Graham Cooke, John Dillon, Charles Gore, Neil Guha, Rachel Halford, Cham Herath, Chris Holmes, Anita Howe, Emma Hudson, William Irving, Salim Khakoo, Paul Klenerman, Diana Koletzki, Natasha Martin, Benedetta Massetto, Tamyo Mbisa, John McHutchison, Jane McKeating, Alec Miners, Andrea Murray, Peter Shaw, Chris C. A. Spencer, Paul Targett-Adams, Emma Thomson, Peter Vickerman, Nicole Zitzmann, Peter Simmonds, Vincent Pedergnana, Santseharay Ramirez, Jens Bukh, Eleanor Barnes, M. Azim Ansari

**Affiliations:** 1 Peter Medawar Building for Pathogen Research, Nuffield Department of Medicine, University of Oxford, Oxford, OX3 1SY UK; 2 Copenhagen Hepatitis C Program (CO-HEP), Department of Infectious Diseases, Hvidovre Hospital and Department of Immunology and Microbiology, Faculty of Health and Medical Sciences, University of Copenhagen, Copenhagen, Denmark; 3 Wellcome Centre for Human Genetics, University of Oxford, Oxford, OX3 7BN UK; 4 School of Life Sciences, École Polytechnique Fédérale de Lausanne, Lausanne, Switzerland; 5 Precision Medicine Unit, University Hospital and University of Lausanne, Lausanne, Switzerland; 6 Swiss Institute of Bioinformatics, Lausanne, Switzerland; 7 MRC-University of Glasgow Centre for Virus Research, Glasgow, G61 1QH UK; 8 Barts Liver Centre, Blizard Institute, Queen Mary University of London, London, UK; 9 NIHR Nottingham Biomedical Research Centre, Nottingham University Hospitals NHS Trust and the University of Nottingham, Nottingham, UK; 10 MIVEGEC, Université de Montpellier, CNRS, 34000 Montpellier, France; 11 University of Nottingham, Queen’s Medical Centre, Nottingham, NG7 2UH UK; 12 Gilead Sciences, Inc., Foster City, CA USA; 13 Conatus Pharmaceuticals, 16745 West Bernardo Drive, Suite 200, San Diego, CA 92127 USA; 14 Wright-Fleming Institute, Imperial College, London, UK; 15 University of Dundee, Ninewells Hospital & Medical School, Dundee, DD1 9SY UK; 16 Hepatitis C Trust, 27 Crosby Row, London, SE1 3YD UK; 17 Gilead Sciences, Stockley Park, 2 Roundwood Avenue, Middlesex, UB11 1AF UK; 18 Department of Statistics, University of Oxford, Oxford, OX1 3LB UK; 19 BC Centre for Excellence in HIV/AIDS, St. Paul’s Hospital, 608–1081 Burrard Street, Vancouver, BC V6Z 1Y6 Canada; 20 University of Southampton, University Road, Southampton, SO17 1BJ UK; 21 Janssen Diagnostics, Turnhoutseweg, 30, 2340 Beerse, Belgium; 22 UC San Diego, La Jolla, CA 92093-0507 USA; 23 Public Health England, 61 Colindale Avenue, London, NW9 5EQ UK; 24 Nuffield Department of Medicine and the Oxford NHIR BRC, University of Oxford, Oxford, OX1 3SY UK; 25 London School of Hygiene & Tropical Medicine, 15-17 Tavistock Place, London, WC1H 9SH UK; 26 OncImmune Limited, Clinical Sciences Building, Nottingham City Hospital, Hucknall Road, Nottingham, NG5 1PB UK; 27 Merck & Co., Inc, Kenilworth, NJ 07033 USA; 28 Medivir AB, Box 1086, 141 22 Huddinge, Sweden; 29 University of Bristol, Oakfield House, Oakfield Grove, Clifton, BS8 2BN Bristol, UK; 30 University of Oxford, South Parks Road, Oxford, OX1 3QU UK

**Keywords:** Antivirals, Hepatitis C virus, Viral infection

## Abstract

Persistent hepatitis C virus (HCV) infection is a major cause of chronic liver disease, worldwide. With the development of direct-acting antivirals, treatment of chronically infected patients has become highly effective, although a subset of patients responds less well to therapy. Sofosbuvir is a common component of current de novo or salvage combination therapies, that targets the HCV NS5B polymerase. We use pre-treatment whole-genome sequences of HCV from 507 patients infected with HCV subtype 3a and treated with sofosbuvir containing regimens to detect viral polymorphisms associated with response to treatment. We find three common polymorphisms in non-targeted HCV NS2 and NS3 proteins are associated with reduced treatment response. These polymorphisms are enriched in post-treatment HCV sequences of patients unresponsive to treatment. They are also associated with lower reductions in viral load in the first week of therapy. Using in vitro short-term dose-response assays, these polymorphisms do not cause any reduction in sofosbuvir potency, suggesting an indirect mechanism of action in decreasing sofosbuvir efficacy. The identification of polymorphisms in NS2 and NS3 proteins associated with poor treatment outcomes emphasises the value of systematic genome-wide analyses of viruses in uncovering clinically relevant polymorphisms that impact treatment.

## Abstract (web summary)

Sofosbuvir is a common therapy in hepatitis C virus infection, which targets the NS5B polymerase. Here, Smith et al. analyze the association between whole genome HCV polymorphisms and sofosbuvir treatment failure and identify three common polymorphisms present in non-targeted NS2 and NS3 proteins associated with reduced treatment response.

## Introduction

An estimated 70 million people worldwide are persistently infected with hepatitis C virus (HCV), a major aetiology of chronic liver disease, which can culminate in cirrhosis and hepatocellular carcinoma^1^. Since 2011, the rapid development of oral direct-acting antivirals (DAA) that target three different HCV non-structural (NS) proteins has resulted in significant improvements in the safety and efficacy of treatments that cure HCV infection (sustained virologic response (SVR)). Combinations of DAAs targeting different HCV proteins regularly achieve SVR rates in excess of 95%^2,3^.

Sofosbuvir is a nucleotide analogue and competitively binds to and blocks the HCV NS5B polymerase and inhibits viral replication^4,5^. Sofosbuvir is a key component in several currently utilised treatment regimens and is recommended for use in combination with other DAAs, such as NS5A inhibitors ledipasvir, daclatasvir or velpatasvir and the NS3 protease inhibitor voxilaprevir. Sofosbuvir in combination with velpatasvir and voxilaprevir has become one of the recommended salvage regimens for patients unresponsive to previous DAA therapies^6,7^. Sofosbuvir is effective against all HCV genotypes and has a high barrier to the development of resistance^8^. However, pre-treatment resistance-associated substitutions (RAS) for both NS3 protease and NS5A inhibitors used in combination with sofosbuvir^9–12^ are commonly reported. Subtypes 3b and 4r have been shown to be inherently resistant to available NS5A inhibitors^13^. Where RAS are present, they can cause a large reduction in the potency of NS3 protease and NS5A inhibitors, making sofosbuvir the main active compound in these regimens.

Only a small number of sofosbuvir-associated RAS have been reported^8,14^ relative to other DAAs. In multiple clinical trials using sofosbuvir, NS5B substitutions L159F, S282T, C316H/N, L320F and V321A are reported as RAS and have been detected post-treatment in patients who do not achieve SVR^14^, although only in a small subset. The acquisition of these RAS has been shown to have a large fitness cost for the virus in vitro^11^ and thus they rarely persist for long after treatment failure^15^. An exception is the S282T RAS, which can persist if compensatory mutations are also acquired^16^. Certain HCV subtypes (4r) have been shown to acquire this substitution more readily^9,10^. Furthermore, with the exception of S282T, all sofosbuvir RAS show negligible increases in resistance using in vitro models^11^ and patients who were unresponsive to sofosbuvir containing regimens, often do not carry any of these RAS at baseline or post-treatment. We recently reported the NS5B A150V polymorphism as conferring resistance to sofosbuvir^17^ using patient data and in vitro assays, although others have shown no effect on sofosbuvir resistance in other in vitro assays^18^. This polymorphism unlike previously reported substitutions is common in pre-treatment HCV subtype 3a sequences and may reduce access to the active site of the HCV polymerase^19^. To date, the mechanism of treatment failure in the small proportion of individuals who do not respond to sofosbuvir-based regimens remains unknown. Most studies of sofosbuvir RAS have focused on NS5B protein and the comparison of the post-treatment to baseline sequences, which can only detect treatment emergent RAS. Investigating the impact of the pre-existing polymorphisms in NS5B and other HCV proteins could provide novel insight into viral determinants of sofosbuvir treatment and help us stratify patients more effectively.

In this work, we use full length HCV genomes and deep-sequencing data from 507 patients with HCV gt3a infection and treated with sofosbuvir as the only DAA (sofosbuvir + ribavirin ± Interferon alpha, see “Methods” for cohort and treatment details) from the BOSON clinical trial^20^, to identify viral determinants of sofosbuvir treatment outcome. We perform a viral genome-wide association study (V-GWAS) of baseline sequences and identify three viral polymorphisms outside the NS5B protein, that are significantly associated with SVR and we refer to as treatment outcome polymorphisms (TOPs). Additionally, we find a stepwise reduction in the SVR rate associated with an increase in the total number of TOPs. The TOPs are also associated with a lower reduction in viral load during the first week of therapy and are enriched in the post-treatment sequences in patients who did not achieve SVR. We perform in vitro short-term sofosbuvir dose-response assays using DBN3acc mutants for each TOP and their combinations but observe a negligible impact on the efficacy of sofosbuvir indicating a mechanism of action possibly independent of drug binding. The approach demonstrates the potential of genome-wide assessment of viral sequences to provide unexpected insight into molecular interactions and their importance in improving treatment of viral infections.

## Results

### Non-viral factors associated with SVR

Baseline whole genome HCV sequences were generated for 568 patients enrolled in the BOSON study^20,21^, 518 of which were gt3, 49 were gt2 and one was a gt1a/2b recombinant virus. HCV is a highly diverse pathogen and to limit the impact of virus population structure on our analysis, we restricted the analyses to samples from patients infected with subtype 3a (*N* = 507). Overall 82% (416/507) of the gt3a patients achieved SVR.

To account for non-viral factors associated with SVR, we used logistic regression to test for association between treatment outcome and patient’s cirrhosis status, gender, baseline viral load, prior IFN-based treatment and *IFNL4* SNP rs12979860 genotypes (CC vs non-CC) in a multivariate model. Cirrhosis status was significantly associated with SVR (*P* = 1.5 × 10^−4^), having the largest impact on treatment response (failure rate in cirrhotic patients 29% = 47/163 and in non-cirrhotic patients 13% = 44/344). Male gender and the non-CC genotype of the *IFNL4* SNP rs12979860 were also significantly associated with increase in treatment failure rate (*P*_gender_ = 0.016, *P*_*IFNL4*_ = 6.4 × 10^−3^). Previous IFN-based treatment and higher viral load were associated with statistically non-significant increases in treatment failure (Supplementary Fig. 1). In all our subsequent regression-based analysis *INFL4* genotype, cirrhosis, gender, previous IFN-based treatment and log10 of baseline viral load were added as covariates to account for possible confounders.

During the BOSON clinical trial, patients were randomised into three treatment arms of sofosbuvir and ribavirin for 16 weeks (16 SOF + RBV), sofosbuvir and ribavirin for 24 weeks (24 SOF + RBV), or sofosbuvir plus ribavirin and peginterferon-alfa for 12 weeks (12 SOF + RBV + IFN). These treatment arms had different SVR rates, but as patients were randomised into these treatment arms (which means viruses were also randomised), the confounding between viral factors and the treatment arms is minimised. We therefore decided not to include treatment arm as a covariate in our analysis as it reduces the power of the tests, but have investigated the association between TOPs and the treatment arms to ensure there is no correlation between the two.

### Previously reported sofosbuvir RAS

We investigated the baseline sequences for presence of previously reported sofosbuvir RAS in the NS5B protein (A150V, L159F, S282T, C316H/N, L320F and V321A)^8,14,22^. Only A150V (*n* = 204) and L159F (*n* = 1) were detected in the consensus sequences (sequence data for these sites was not available for six patients). A150V was significantly associated with outcome (*P* = 4.92 × 10^−3^) and patients carrying valine at this site had an SVR rate of 75% (*n* = 153/204) vs. 86% (*n* = 257/297) for patients with other amino acids at this site. We have previously reported this association in a subset of this cohort and showed it reduces sensitivity to sofosbuvir using in vitro assays^17^. We have also previously reported an association between this viral site and host *IFNL4* SNP rs12979860^21^ genotypes. Stratifying the patients based on the *IFNL4* SNP rs12979860 genotypes (CC vs. non-CC (genotyping data not available for two patients)), we observed that, among CC patients, the impact of valine on SVR rate was minimal (SVR_valine_ 85% = 35/41, SVR_non-valine_ 87% = 129/148, Fisher’s exact test P = 0.80). On the contrary, among non-CC patients it reduced SVR rate substantially (SVR_valine_ 72% = 117/162, SVR_non-valine_ 85% = 127/148, Fisher’s exact test *P* = 0.0036). However, the interaction term was statistically nonsignificant (*P* = 0.36).

Analysis of minor populations in the NGS data revealed very low frequencies (<1% of reads) of S282T (two patients), C316N (one patient), L320F (two patients) and V321A (eight patients).

### Identification of TOPs using viral genome-wide association study (V-GWAS)

A major advantage of HCV whole-genome sequencing is the possibility of testing viral genetic variants for associations with SVR across the entire viral genome to identify potentially novel associations between HCV genetic variants and response to sofosbuvir treatment. Initially, we investigated whether any of the HCV lineages were associated with treatment outcome. To do this we built a maximum likelihood phylogenetic tree (Supplementary Fig. 2) from baseline consensus sequences. Analysis by treeBreaker software^23^ did not reveal any clades that had a different SVR rate from the rest of the tree (Bayes factor = 0.969, see “Methods”).

**Fig. 1:**
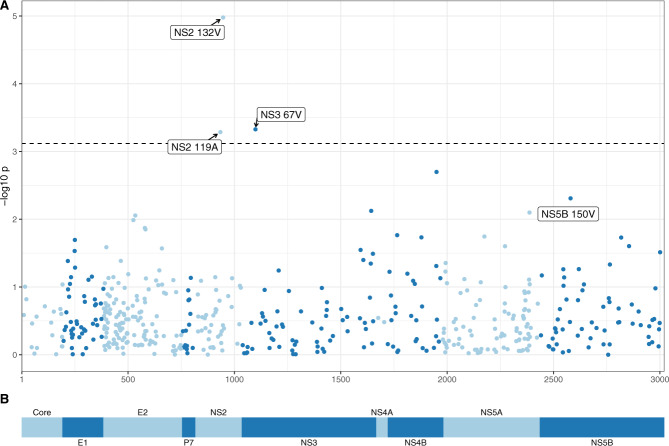
Association between hepatitis C virus (HCV) amino acids and sofosbuvir-based treatment outcome. **A** Manhattan plot of association tests between HCV amino acids and treatment outcome. At each viral site only the p-value for the most associated amino acid is plotted. The dashed line indicates 15% false discovery rate. For the three sites significantly associated with outcome and the most associated site in the non-structural 5B (NS5B) protein, the amino acid associated with the lowest cure rate at the site and its position within its respective protein is indicated. **B** Schematic of HCV polyprotein.

We then performed a V-GWAS, using logistic regression to test for association between sofosbuvir treatment outcome and encoded amino acids at each site, including the non-viral factors previously indicated as covariates. The first three viral genetic principal components (PCs) and the first three host genetic PCs were also included as covariates to account for host-virus population co-structuring. In our analysis, SVR status was used as the response variable and the presence and absence of each amino acid as the explanatory variable. We only tested amino acids that were present in sequences from at least 20 individuals; this resulted in 1010 tests at 484 sites. At a false-discovery rate (FDR) of 15%, three viral polymorphisms were significantly associated with SVR (Fig. 1 and Supplementary Table 1, the numbers below may not add to 507 as not all sites on the viral polyprotein were available for all samples, see Supplementary Table 1). The most significant association with SVR was at position 132 in the NS2 protein (*P* = 1.05 × 10^−5^), where isoleucine (80% = 405/506) and valine (20% = 100/506) alternated (one isolate carried leucine). Valine was associated with a reduction in treatment response (SVR 66% = 66/100) relative to the isoleucine residue (86% = 348/405). Two additional significant associations with reduction in SVR rate were valine at position 67 in the NS3 protein (*P* = 4.71 × 10^−4^) and alanine at position 119 in the NS2 protein (*P* = 5.18 × 10^−4^) (Fig. 1, Supplementary Table 1 and Supplementary Table 2). In the NS5B protein (the direct antiviral target of sofosbuvir), valine at position 150 was ranked first (lowest *P*-value, *P* = 4.92 × 10^−3^) although the association was not significant genome-wide at a 15% FDR. For the remainder of this work NS5B A150V is included as a TOP.

Next we used an alternative chronic HCV infection cohort of 144 patients provided by HCV Research UK to replicate the findings. All patients had cirrhosis and 107 (75%) were treated with sofosbuvir in combination with an additional NS5A inhibitor such as daclatasvir, ledipasvir or velpatasvir, while the remaining 37 (25%) patients were treated either with SOF + RBV or SOF + RBV + INF. In this replication cohort, only NS2 119A was nominally associated with SVR (*P* = 0.045). Overall, the effect size estimates were similar for three of the four TOPs (NS2 119A, NS2 132V and NS5B 150V), but they had larger confidence intervals due to smaller samples sizes and additional DAAs used in the therapy (Supplementary Fig. 3).

### Covariation of TOPs and their impact on sustained virologic response

**Fig. 2:**
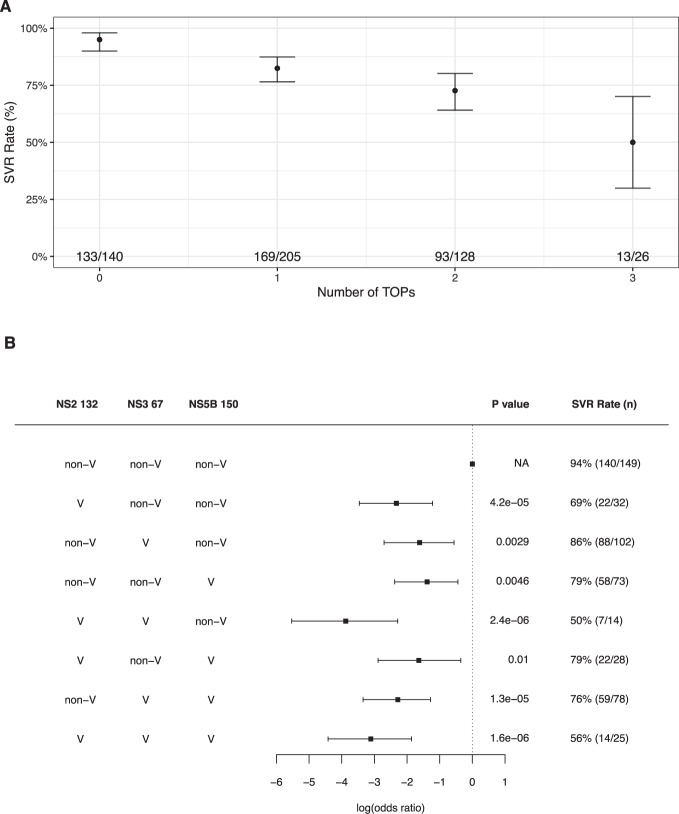
Covariation of treatment outcome polymorphisms (TOPs) and their impact on sustained virological response (SVR) rate. **A** SVR rate for patients with different numbers of TOPs in baseline sequences. The dots indicate the SVR rate in each group and the lines indicate its 95% confidence intervals. The numbers in each group are shown at the bottom of the figure. **B** Covariation between the three novel TOPs and their association with treatment outcome. Combinations tested are listed in the table on the left. The squares show the estimated effect size (log(odds ratio)) for each group and the lines show its 95% confidence interval estimated from logistic regression. The *P*-value (logistic regression) and the SVR rate for each group are shown on the right.

To understand the impact of presence of multiple TOPs in each sequence on SVR, we stratified patients based on the number of TOPs present in their baseline consensus sequences (Fig. 2A). The SVR rate for patients whose virus carried no TOPs was 95% (133/140), which was reduced to 50% (13/26) for patients with three TOPs. Only one patient carried all four TOPs and this patient achieved SVR. The step wise reduction in the SVR rate associated with the increase in the number of TOPs present at baseline was highly significant (logistic regression *P* = 6.6 × 10^−10^).

We also investigated the covariation between these TOPs and their impact on SVR. NS2 119A was excluded from this analysis because of its low frequency (4% = 22/505). We tested for association (covariation) between the three pairs using logistic regression, including the first three viral PCs and host *IFNL4* SNP rs12979860 genotypes (CC vs. non-CC) as covariates to account for virus population structure and the impact of *IFNL4* on the HCV amino acids. NS5B 150V was significantly associated with NS2 132V (*P* = 0.004) and nominally with NS3 67V (*P* = 0.02). NS2 132V and NS3 67V were not significantly associated with each other (*P* = 0.83). Stratifying the patients by presence and absence of TOPs (valine residue) at the three sites of NS2 132, NS3 67 and NS5B 150, we observed that patients carrying viruses with non-valine residues at all three sites had the highest SVR rate (94% = 140/149), while carrying any one of these TOPs individually or in combination reduced SVR rate significantly (Fig. 2B). Carrying valine at both NS2 132 and NS3 67 sites, but not NS5B 150 was associated with the lowest SVR rate of 50% (*P* = 2.4 × 10^−6^). We also tested for non-additive interaction effects on SVR between the three sites and found that the only significant non-additive interaction effect was between NS2 132 and NS5B 150 sites (interaction *P* = 0.01) where carrying valine at both sites resulted in SVR recovery (79% = 22/28) rather than an additive reduction.

### TOPs effects on viral load reduction during first week of therapy

**Fig. 3:**
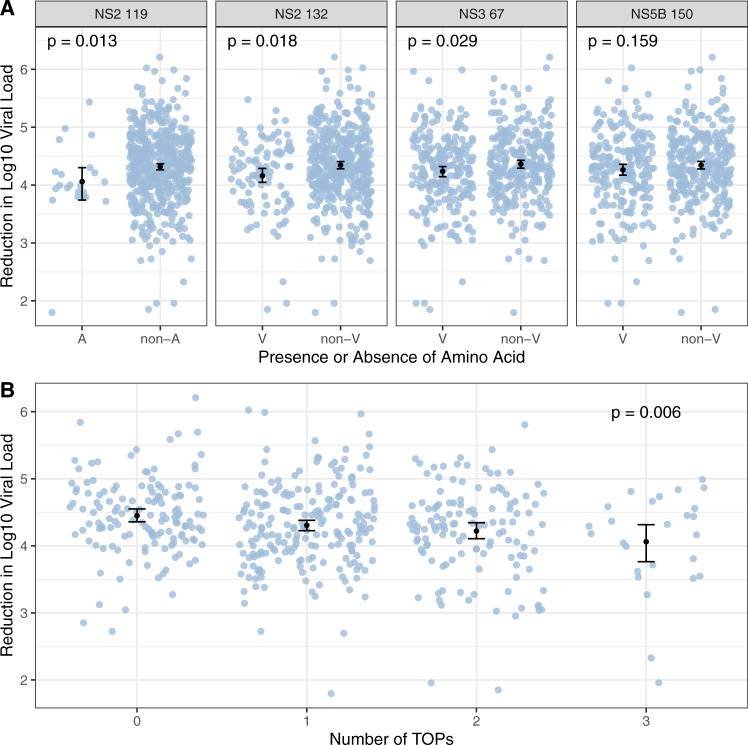
Reduction in viral load during the first week of therapy and its association with treatment outcome polymorphisms (TOPs). **A** Reduction in log10 viral load between baseline and week one of therapy stratified by presence and absence of the residue associated with the lowest SVR rate at each TOP (A: Alanine, non-A: any amino acid other than alanine, V: Valine, non-V: any amino acid other than Valine). *n* = 507 patients. The mean change in viral load is shown as a black dot for each TOP and the lines indicate its 95% confidence intervals. *P*-values for difference in mean calculated using one-sided Mann–Whitney test. **B** Reduction in log10 viral load between baseline and week one of therapy against increasing numbers of TOPs (presence or absence of residue associated with the lowest SVR rate at the target sites). *n* = 507 patients. The mean change in viral load is shown as a black dot and the lines indicate its 95% confidence interval. *P*-value for association between number of TOPS and reduction in log10 viral load calculated using linear regression.

If these TOPs reduce the impact of treatment, one expects to observe differences in the reduction in viral load during therapy between patients with and without these TOPs. Thus, we evaluated the impact of these novel TOPs on the viral load reduction from baseline to week one of therapy and tested for difference in mean reduction between patients with and without each TOP and also their combination (Fig. 3). At each of the sites, the mean reduction in viral load was smaller for TOPs relative to non-TOPs residues. The reduction in viral load was nominally significant for patients whose virus carried TOPs at sites NS2 119 (*P* = 0.013), NS2 132 (*P* = 0.018) or NS3 67 (*P* = 0.029) relative to those whose virus did not carry the TOPs at these sites. The impact of the total number of TOPs carried by each patient on the viral load reduction was also significant (linear regression, *P* = 0.006, Fig. 3B). Investigating the eight possible combinations of the three sites of NS2 132, NS3 67 and NS5B 150 we observed that the presence of valine at the two sites of NS2 132 and NS3 67 and non-valine at NS5B 150 resulted in the lowest reduction in viral load during therapy (*P* = 0.00091, Supplementary Fig. 4). We also tested for association between the TOPs and the baseline viral load (log10 viral load). The NS2 119 A TOP was nominally associated with a higher viral load (*P* = 0.029, linear regression), none of the other TOPs were associated with baseline viral load.

### Prevalence and enrichment of TOPs in post treatment sequences

Ninety-one patients in this trial did not achieve SVR and we were able to generate 73 whole-genome consensus sequences from plasma samples taken at 12 weeks post-treatment (12WPT). These consensus sequences were scanned for the presence of previously reported sofosbuvir RASs. NS5B 159F was detected in four isolates. This represents a significant change in the distribution of this RAS in the post treatment sequences relative to the baseline sequences (binomial test, *P* = 1.65 × 10^−5^). The NS5B RAS 282T, 316H/N, 320F and 321A were not detected in any of the post treatment consensus sequences. On analysis of the quasi-species data the 159F was detected in two additional 12WPT samples as minor variant (<15% of reads). The 321A was detected at low frequencies (<1% of reads) in two samples.

We also investigated the distribution of the TOPs identified using baseline sequences in our study in the 12WPT sequences. A binomial test was performed to identify changes in the distribution of the TOPs at these sites between the baseline population and the 12WPT sequences (Table 1). NS5B 150V was significantly enriched in the 12WPT sequences (*P* = 0.00088) and NS2 132V (*P* = 0.019) and NS3 67V (*P* = 0.023) were nominally significantly enriched. The majority of these polymorphisms were present at baseline and were carried forward to the post-treatment isolates, suggesting that the increase in prevalence is due to enrichment of pre-existing polymorphisms rather than substitutions during therapy. However, this was not the case for the previously reported NS5B 159F RAS, where three of the four RAS had developed during therapy.

**Table 1:** Prevalence of resistance associated substitutions (RASs) and treatment outcome polymorphism (TOPs) at baseline and 12 weeks post treatment (12WPT).

Protein	RAS/TOP	Prevalence at baseline (*n*)	Prevalence at 12WPT (*n*)	*P-*value	Proportion of RAS/TOP at 12WPT not present at baseline (*n*)
Previously characterised RAS					
NS5B	150V	41% (204/501)	60% (43/72)	8.77 × 10^−4^	9% (4/43)
NS5B	159F	0% (1/501)	6% (4/72)	1.47 × 10^−5^	75% (3/4)
TOPs					
NS2	119A	4% (22/505)	8% (6/72)	9.37 × 10^−2^	17% (1/6)
NS2	132V	20% (100/506)	30% (22/72)	1.94 × 10^−2^	0% (0/21)
NS3	67V	44% (222/506)	56% (41/73)	2.33 × 10^−2^	7% (3/41)

The percentage of the population with each RAS or TOP is shown at baseline and 12WPT. These distributions were compared using a one-sided binomial test and the *P*-value is shown (nominal *P*-value, not adjusted for multiple comparison). The percentage of patients where the amino acid at baseline has changed to the RAS or TOP in post-treatment sequence is also shown.

### In vitro evaluation of sofosbuvir antiviral activity against TOPs using a cell culture adapted genotype 3a isolate (DBN3acc system)

We investigated the effect of the TOPs using short-term dose-response sofosbuvir assays, which reflect drug binding. The cell culture adapted genotype 3a DBN3acc virus was mutated using site directed mutagenesis^18,24^ to contain individual and combinations of TOPs (see Methods); a full list of the mutants tested can be found in Table 2. All the TOP containing mutants were viable after transfection except for the combination of NS2 119A, NS2 132V and NS5B 150V. The NS2 132V mutant exhibited a slight delay in viral spread after transfection, whereas the remaining mutants propagated and produced infectious viruses similar to the original cell culture adapted DBN3acc virus (Table 2). All viruses were genetically stable after passage, maintaining the introduced mutations. We did observe the emergence of a population harbouring two extra mutations (A4506A/G (amino acid I1389I/M) in NS3 and A7480A/G (amino acid T2381T/A) in NS5A) in the DND3acc NS2 132V virus (present at 50% together with the original residue as indicated in Sanger sequencing). None of the differences in the EC_50_ between mutants and the original virus reached a 2-fold threshold, which we considered as a minimum to be recognised as providing decreased activity of sofosbuvir in this particular assay (Table 2, dose response curves can be found in Supplementary Fig. 5).

**Table 2:** Characteristics of HCV DBN3acc mutants after transfection and subsequent passage in Huh7.5 cells.

Mutant	Spread (day)^a^	Infectivity titre (day)^b^	Fold Change EC_50_^c^	Engineered mutations
DBN3acc	10	4.65 (10)	N/A	Maintained
NS2 119A	10	4.71 (10)	1.02	Maintained
NS2 119V	10	4.76 (10)	1.22	Maintained
NS2 132V	17	4.10 (23)	0.61	Maintained^d^
NS3 67A	10	4.49 (10)	0.65	Maintained
NS5B 150V	10	4.50 (14)	1.00	Maintained
NS2 119A + NS2 132V	10	4.62 (14)	1.14	Maintained
NS2 119V + NS2 132V	10	4.33 (14)	0.60	Maintained
NS2 119A + NS3 67A	10	4.51 (10)	1.19	Maintained
NS2 119V + NS3 67A	10	4.40 (10)	1.06	Maintained
NS2 119V + NS2 132V+ NS3 67A	10	4.56 (14)	0.98	Maintained
NS2 119A + NS2 132V + NS5B 150V^e^	N/A	N/A	N/A	N/A
NS2 119V + NS2 132V + NS5B 150V	10	4.39 (14)	0.98	Maintained
NS2 119A + NS3 67A + NS5B 150V	10	4.14 (14)	0.79	Maintained

Viral kinetics, sofosbuvir susceptibility and genetic stability of DBN3acc original and mutant viruses.

^a^Day of spread after transfection, which corresponds to the day where ≥80% HCV antigen-positive cells were observed in the culture.

^b^The highest infectivity titre observed in supernatants harvested after transfection with the day indicated in parenthesis. Data is based on the mean of three replicates and the titre units are Log10 focus-forming units per millilitre (FFU/ml).

^c^Change in sofosbuvir susceptibility shown as fold-change in EC50 values between original and mutant viruses.

^d^The DBN3acc-NS2I132V mutant virus acquired substitutions A4506A/G (aa I1389I/M in NS3) and A7480A/G (aa T2381T/A in NS5A) as detected during passage.

^e^Recombinant DBN3acc- NS2 119A + NS2 132V + NS5B 150V was non-viable in two independent transfections. N/A: not applicable.

### Association of TOPs and host HLA alleles

We examined the association between the TOPs and the host HLA alleles and their restricted epitopes. The TOPs NS2 119A and NS2 132V are both found within previously identified HCV gt3a T-cell epitopes^25^. To investigate if the host HLA alleles can explain the TOPs association with treatment outcome, first we investigated if any of the HLA alleles were associated with treatment outcome. We used logistic regression with the same covariates as in the V-GWAS to test for association between the HLA alleles and SVR. To retain power, we only tested at the two-digit level and only tested HLA alleles present in at least 20 individuals. At 20% FDR no HLA allele was significantly associated with treatment outcome (Supplementary Table 3). Next, we tested for association between the TOPs and the HLA alleles using the same logistic regression model (Supplementary Table 3). At 20% FDR the only association observed was between HLA B*55 and NS2 132V. To understand if the impact of the NS2 132V on SVR is mediated through its association with HLA B*55, we tested for association between SVR and NS2 132V using logistic regression conditioning on HLA B*55 (including it as a covariate in the model). Conditioning on HLA B*55 had negligible impact on the association between NS2 132V and SVR (original *P* = 1.05 × 10^−5^, model including B*55, *P* = 1.17 × 10^−5^).

## Discussion

In this study, we report the first systematic and comprehensive investigation of the viral determinants of treatment outcome in a large cohort of HCV gt3a infected patients treated with SOF + RBV ± /−INF. We generated HCV whole genomes using next generation sequencing technologies for 507 patients at baseline and 73 patients who failed therapy at 12WPT. Using V-GWAS we performed a systematic assessment of the impact of HCV amino acids across all proteins on sofosbuvir treatment outcome. In this study we report for the first time, amino acid polymorphisms in HCV NS2 and NS3 proteins associated with treatment outcome. We also examined combinations of TOPs, the total number of TOPs carried, reduction in viral load during therapy and post-treatment failure sequences for additional evidence of the impact of these novel TOPs. We expect that with advances in high-throughput virus whole genome sequencing and the reduction in cost^26^, analysis of this kind will become a powerful tool for understanding the viral genetic variants driving treatment response and whether stratification of patients is required based on viral genetics to optimise treatment outcomes.

Traditionally, RAS have been identified in vivo by comparing baseline sequences to post treatment failure sequences in the protein targeted by the drug^27^. However, virus is usually undetectable in plasma for several weeks after the end of the treatment and the RAS with high fitness cost to the virus are likely to have reverted back to the wild type shortly after the end of the treatment. RAS are also identified in vitro by performing viral selection studies. The majority of RAS have been tested using in vitro assays to determine their impact on viral replication in cell culture^16^. This method has worked well for protease inhibitors and NS5A inhibitors where there is a strong corelation between in-vivo and in-vitro data. However, for nucleotide analogues like SOF this method has been less successful. Any mutations that reduces the binding of the drug such as NS5B S282T will also have a high fitness cost to the virus^28^. The loss off fitness may be reduced by existing TOPs that may reduce fitness cost of the transient mutations, which reduce the effect of the nucleotide analogue. This process is difficult to identify from sequencing treatment failures due to the transient nature of the mutations, which provide resistance. It is also difficult to replicate this using only in-vitro models due to the complex interactions between HCV proteins essential to the viral life cycle^29,30^ and the fact the cell culture models do not represent the physiological and pathological conditions of a human liver^28,29^. Instead a combined approach combining viral sequence analysis such as V-GWAS and in-vitro analysis is required to identify the viral factors, which cause nucleotide analogue resistance.

With the benefit of full-length HCV genomic data enabling a comprehensive analysis of all HCV proteins, we have found strong evidence that common polymorphisms in HCV NS2 and NS3 proteins modify treatment outcome in patients receiving SOF + RBV ± /−INF. The treatment arm including interferon-alpha had the highest SVR rate and therefore the mechanism of action of the TOPs is unlikely to be associated with interferon-alpha. Although several mechanisms of action for ribavirin have been proposed^31^, a main antiviral effect for HCV can be achieved through genome-wide mutagenesis^18^, however direct effects on HCV RNA levels are not observed in patients on ribavirin monotherapy^32^. The hereby identified TOP sites have not been reported in previous studies characterising ribavirin resistance^33^. Whilst two of the TOPs were found to be in previously reported HCV T Cell epitopes^25^, we did not observe a significant association between SVR and any HLA alleles. At 20% FDR, we found an association between NS2 132V and HLA B*55. However, when accounting for the HLA B*55, the impact of NS2 132V on SVR did not change.

A plausible mechanism of action of these TOPs is that they may reduce the antiviral effect of sofosbuvir by directly affecting the viral life cycle in the presence of sofosbuvir, for instance modulating replication and assembly or by directly affecting drug interactions in the replication complex, and therefore influencing treatment outcome. However, our in vitro cell culture analysis of TOPs demonstrated no evidence of increase in the sofosbuvir EC_50_ in short-term dose-response assays, which suggests no significant changes in drug affinity. This observation agrees with prior studies showing that even putative sofosbuvir RASs in the NS5B protein do not change the EC_50_ of sofosbuvir unless the S282T substitution is present^34^. Thus, even if TOPs do not directly affect sofosbuvir binding, they may enhance the virus ability to overcome the inhibition of sofosbuvir by increasing overall viral fitness in the presence of the drug or by promoting the transient emergence of mutations (such as S282T) that will decrease drug binding, these may disappear before the patients viral load is high enough for detection via sequencing, as they cause a high fitness cost. This effect may be more evident in less potent treatments such as SOF + RBV ± INF and can efficiently be counteracted in pan-genotypic regimens that include several potent DAA that can more rapidly inhibit viral replication. Another study has also identified sites outside the NS5B associated with sofosbuvir-based treatment outcome, including sites in similar regions of the NS2 and NS3 protein^35^. However, this was in gt1a HCV and in a cohort with only 20 patients.

Sofosbuvir targets the NS5B viral RNA polymerase. Although NS2 and NS3 proteins are not directly targeted by sofosbuvir, HCV replication and production are critically dependent on complex interactions between the viral proteins. HCV NS2 is a membrane-bound autoprotease that is required for cleavage at the NS2/NS3 boundary^36–39^. Autoprotease activity is enhanced by the N-terminal region of NS3^36^. Although NS2 is dispensable for HCV RNA replication, the release of NS3 from the polyprotein by NS2 is essential for viral RNA synthesis to proceed. Aside from its autoprotease activity, NS2 plays an essential and central role in assembly of infectious virus, which requires complex interactions with both structural and non-structural proteins^40^. The N-terminal one-third of NS3 also possesses protease activity while the remaining two-thirds encode a RNA helicase^41^. The position of the identified TOPs lies within the protease domains of both proteins^42,43^. In NS2, amino acid position 119 lies in a loop region between two anti-parallel α-helices H1 and H2 while residue 132 is located in α-helix H2^42^. It has been proposed that the loop containing residue 119 may lie close to or interact with a cellular membrane^42^. Position 67 in the NS3 protease domain sits in a loop region between two beta sheets^43^. The functional significance of the variants at these positions in NS2 and NS3 on the virus life cycle is not obvious. Nonetheless, data from in vitro studies have demonstrated that tissue culture adapted variants in HCV proteins that include NS2, NS3 and NS5B can modulate virus production as well as interactions between the viral proteins. Thus, there is the potential for variants to arise that could modify the complex protein–protein interactions needed for both HCV RNA replication and virion assembly, and thereby promote DAA resistance in proteins that are not obvious targets for the drug^44^.

By stratifying the patients, we observed that the reduction in SVR rate based on the combination of these TOPs was broadly additive with patients having both NS2 132V and NS3 67V having an SVR rate of 50% vs. 69 and 86% for patients carrying only NS2 132V or NS3 67V TOP, respectively. Patients with viruses that did not carry any TOP had an SVR rate of 94%. We also observed a step-wise reduction in SVR rate as the total number of TOPs present in baseline sequences increased. In studying the impact of these TOPs on reduction in viral load from baseline to week one of therapy, all four TOPs on average resulted in smaller reductions in viral load and this effect was nominally significant for the three sites in NS2 and NS3 proteins. As viral load is a surrogate for viral replication and virion production, its reduction during therapy is an independent measure that can highlight impact on treatment.

We studied the impact of these novel TOPs in an independent cohort of patients treated with sofosbuvir in combination with other DAAs regimes such as NS5A inhibitors (daclatasvir or ledipasvir). The direction and estimated effect sizes were consistent for three of the four sites. However, the associations were not significant as the confidence intervals were larger. It is important to note that this is not an equivalent replication cohort due to its small size (144 patients) and the fact that majority of patients were treated with other DAA in combination with sofosbuvir, which will reduce the power for detecting associations. Additionally, therapy including sofosbuvir as the only DAA is no longer recommended and a cohort similar to BOSON is unlikely to be available in the future.

In conclusion, our data show that common HCV gt3a polymorphisms in NS2 and NS3 proteins are associated with sofosbuvir treatment outcome. Assessing these polymorphisms may be useful in directing therapy length and combinations for difficult to treat patients such as those with advanced cirrhosis or those who have failed DAA therapy previously. Patients carrying both NS2 132V and NS3 67V TOPs had the lowest SVR rate (50%) and the smallest reduction in viral load during the first week of therapy. Patients with these polymorphisms may require special attention if treated using sofosbuvir containing therapies. Sofosbuvir is only used in combination with NS5A inhibitors for which RAS are common in baseline HCV sequences. In such cases polymorphisms that reduces the impact of sofosbuvir will be important in deciding the length and the combination of drugs especially in difficult to treat groups.

## Methods

### Patients and samples

#### BOSON clinical trial

Our study is based on the analysis of samples from the BOSON clinical trial^20^ (registration number: NCT01962441) and permission was granted for the use of samples for further studies. Plasma Samples were obtained at baseline, during treatment and 12 weeks post treatment. The patients were randomised into three treatment arms of 12 Weeks SOF + RBV + IFN, 16 weeks SOF + RBV and 24 weeks SOF + RBV. All patients were DAA treatment-naïve. Each patient’s HCV viral load was measured at baseline, week 1, week 2, week 4 and week 8. All patients provided written informed consent before undertaking any study-related procedures. The BOSON study protocol was approved by each recruiting institution’s review board or ethics committee before study initiation. The study was conducted in accordance with the International Conference on Harmonisation Good Clinical Practice Guidelines^45^ and the Declaration of Helsinki. Sample sizes were determined by the available data.

#### Replication cohort

The results of the V-GWAS were replicated using whole HCV genome sequences from 144 patients from an independent cohort (HCV Research UK cohort^46^). All patients provided written informed consent before undertaking any study-related procedures, The use of data for this study was approved by the HCV Research UK Tissue and Data Access Committee. All patients were treated with SOF containing regimens. As direction of effects were estimated in the primary cohort, one-sided tests were used to replicate the results in this cohort. Treatment outcome was the response variable and presence/absence of the TOP the explanatory variable. All the patients were cirrhotic and host genotyping data was not available. Therefore, we did not include any covariates in this analysis.

### Viral sequencing

RNA was extracted from 500 μl of plasma using the NucliSENS^®^ easyMAG system (bioMérieux) into 30 μl of water, of which 5 μl was processed with the NEBNext® Ultra™ Directional RNA Library Prep Kit for Illumina® (New England Biolabs) with previously published modifications to the manufacturers protocol^47^. A 500 ng aliquot of the pooled library was enriched using the xGen® Lockdown® protocol from IDT (Rapid Protocol for DNA Probe Hybridisation and Target Capture Using an Illumina TruSeq® Library (v1.0), Integrated DNA Technologies) with a comprehensive panel of HCV-specific 120 nucleotide DNA oligonucleotide probes (IDT), designed using a previously published algorithm^48^. The enriched library was sequenced using Illumina MiSeq v2 chemistry to produce paired 150b reads. Reads were demultiplexed low-quality reads were trimmed with QUASR(v7.0120) and adapter sequences removed using Cutadapt(v1.7.1). Host derived sequences were removed using Bowtie(v2.2.4). HCV reads were selected by mapping against the 162 ICTV (International Committee on the Taxonomy of Viruses) reference sequences for mapping against the closest reference and de novo assembly (Vicuna(v1.3)), read mapping (MOSAIK(v2.2.28)), genome annotation (VFAT(v1.0)) and interpretation of variants (genewise2, Vphaser(v.2.0), Vprofiler(v1.0)). HCV subtype was assigned based on the sequence similarity to ICTV reference sequences, a phylogenetic tree of BOSON sequences and some ICTV reference sequences can be found Supplementary Fig. 6.

### Statistical methods

All statistical analysis was performed using the statistical package R(v3.6.1), all the analysis code and data is available on request from the STOP-HCV consortium (www.stop-hcv.ox.ac.uk).

#### Non-viral factors associated with outcome

To ensure we accounted for possible confounders when investigating viral effects on outcome, a multivariate analysis was performed using logistic regression to identify non-viral factors, which are associated with SVR. The patient’s cirrhosis status, IFNL4 genotype, prior treatment status, gender, log of baseline viral load and age at time of treatment were investigated as possible confounders. Akaike Information Criterion was used to choose the set of covariates to include in the model. The resulting model (Supplementary Fig. 1) included, patient’s cirrhosis status, IFNL4 genotype, prior treatment status, gender and log of baseline viral load as covariates in subsequent regression-based analysis unless otherwise stated.

#### Previously reported sofosbuvir RAS effect on outcome

The effect of previously reported sofosbuvir RAS present before treatment was investigated using logistic regression. The response variable was treatment outcome (SVR), the explanatory variable was the presence of RAS and the previously discussed non-viral factors associated with outcome were included as covariates along with the first three viral sequence and host genotyping principle components.

#### Phylogenetics and association of a phenotype with tree structure

RAxML(v8.0)^49^ was used to build a maximum likelihood phylogenetic tree using the HCV full genome sequences assuming the general time reversible model of nucleotide substitution under the gamma model of rate heterogeneity. The treeBreaker(v1.0)^23^ software was then used to investigate if SVR or any novel TOP are associated with specific clades on the tree. If any clades were associated with any of the novel TOP, then the logistic regression was repeated adding an indicator variable for the samples under the clade as a covariate, to confirm that the virus population structure was not confounding the association.

#### Viral genome-wide association study

To investigate the effect of individual viral amino acid polymorphisms on SVR a GWAS was performed using a logistic regression model. In our analysis, SVR status was used as the response variable and the presence and absence of each amino acid as the explanatory variable, the non-viral factors previously indicated were included as covariates. To ensure that host-virus population co-structuring had minimal impact on our analysis, we limited our cohort to patients infected with HCV gt3a. To control for both host and virus population structures we included the first three viral PCs calculated from viral sequences and the first three host PCs calculated from the host genome-wide genotyping data as covariates in our model.

#### Covariation and interaction of TOPs analysis

Logistic regression was used to test for association between the number of TOPs in a patient’s viral sequence and SVR. The response variable was SVR and the explanatory variable was number of TOPs in the sequence, the non-viral factors previously indicated were included as covariates. We used logistic regression to investigate possible covariation of TOPs and the first 3 viral PCs and IFNL4 genotype (CC/non-CC) were added as cofounders as IFNL4 has been identified as having an effect on the viral genome. Interaction was tested using logistic regression using interaction terms and the confounders identified in Supplementary Fig. 1.

#### Initial response to therapy

To test if any TOP were associated with a smaller reduction in VL at the start of therapy. The Log10 reduction in viral load from BL to week1 of therapy was calculated. Wilcoxon tests were used to test if patients with TOPs had a lower reduction in viral load than those who did not. Linear regression with the confounders identified in Supplementary Fig. 1 included was used to test for an association between the number of TOPs in a sequences and reduction in VL.

#### Enrichment of an amino acid in the post-treatment HCV sequences

To test for enrichment of an amino acid in the post-treatment samples (that did not achieve SVR), we used a one-sided binomial test. For each amino acid, the frequency of the amino acid in the baseline sequences, the total number of post-treatment sequences and the observed number of the amino acid in the post-treatment sequences was used to test for its enrichment in the post-treatment sequences.

### In vitro testing

#### Cell culture and generation of DBN3acc mutant viruses

All cell culture experiments were performed in human hepatoma cells (Huh7.5 clone) as described using the genotype 3a cell culture adapted DBN3acc system^24^. Mutations were introduced in the DBN3acc plasmid using a megaprimer approach with primers described in Supplementary Table 4 at a final concentration of 10 µM, 50 ng of plasmid template, and the Q5 High-Fidelity 2X Master Mix reagent. For the first PCR cycling conditions were 98 °C for 30 s, followed by 35 cycles of: 98 °C for 10 s, 71 °C for 15 s, and 72 °C for 1 min, followed by a final extension at 72 °C for 5 min. The PCR products were treated overnight with DpnI (NEB) at 37 °C and gel purified using the Zymoclean Gel DNA Recovery Kit (Zymo Research). 200 ng of the amplicon were used as primer in a second PCR reaction (megaprimer reaction) with 50 ng of plasmid template conducted at 98 °C for 30 s, followed by 20 cycles of: 98 °C for 10 s, 48 °C for 1 min, and 72 °C for 20 min, followed by a final extension at 72 °C for 20 min. The PCR products were treated overnight with DpnI (NEB) and 2 µl of product was transformed in XL10-Gold ultracompetent cells (NEB) following the manufacturer’s protocol. All plasmid preparations (Qiagen) were confirmed by Sanger sequencing.

Plasmids were linearised with XbaI (NEB) and treated with Mung Bean nuclease (NEB) prior to in vitro transcription (IVT). IVT were performed using 2 µg of template using the MEGAscript T7 Transcription Kit (Thermo Fisher Scientific) and following the manufacturer’s instructions. RNA IVT transcripts were purified using the RNeasy mini Kit (Qiagen). Transfections were performed with lipofectamine 2000 (Thermo Fisher Scientific) and cells were seeded 24 h prior to the experiment in 6-well plates (5 × 10^5^ cells per well). In brief 5 µl of diluted lipofectamine in 245 µL of Optimem (Thermo Fisher Scientific) was mixed with 2.5 µg of viral RNA also diluted in Optimem (final volume of 250 µl). The RNA/lipofectamine mix was incubated at room temperature for 20 min and added to cells containing Optimem. After 4 h of incubation at 37 °C, the media was replaced by regular culture media (DMEM (high glucose, GlutaMAX and pyruvate, Invitrogen ThermoFisher) supplemented with 10% fetal bovine serum (Sigma), 100 units/ml of penicillin and 0.1 mg/ml of streptomycin/ml [Sigma]). Cultures were split every 2–3 days and virus containing supernatant was harvested, filtered and stored at −80 °C. The viral infection was monitored by immunostaining of HCV viral antigens with mouse monoclonal [C7-50] anti-core (#ab2740, Abcam) and mouse monoclonal 9E10 anti-NS5A antibodies^24,50^ followed by addition of Goat anti-Mouse IgG (H + L) Highly Cross-Adsorbed Secondary Antibody, Alexa Fluor Plus 488 (#A32723, Invitrogen) and Hoechst counter-stain (#33342, Life Technologies)^24^. The number of HCV antigen protein-positive cells were evaluated by fluorescence microscopy (Axio Vert.A1; Zeiss, Jena, Germany). One millilitre of supernatant harvested at days 14 or 17 post-transfection was used for passage of naïve Huh7.5 cells. Supernatants harvested at day 8 post-infection were used for the analysis of the HCV viral sequence (complete ORF)^51^. Extraction and purification of viral RNA was performed using Trizol™ LS (Life technologies), chloroform (Sigma) and the RNA clean & concentrator™−25 kit (Zymo research) following manufacturer’s instructions. Synthesis of cDNA was performed with the Maxima H minus reverse transcriptase system (Thermo Scientific), supplemented with RNasin plus RNase inhibitor enzyme (Promega) and with reverse primer 5′-AAAAGAATGGAGTGTTATC-3′. cDNA was treated with RNase H (Takara) and RNase T1 (Thermo Scientific) and immediately used as template for the PCR amplification. A single amplicon encompassing the entire viral ORF (from nt.293 to nt.9432, [DBN3acc numbering and primers 5′-GATAGGGTGCTTGCGAGTGCC-3′ and 5′-AGAATGGAGTGTTATCCTACCAGCTCA-3′]) was obtained using the Q5® Hot Start High-Fidelity DNA polymerase system (NEB) with PCR cycling conditions as follows: 1 cycle of 98 °C/30 s; 35 cycles of 98 °C/10 s, 65 °C/10 s and 72 °C/8 min; and 1 cycle of 72 °C/1 min. Purified PCR products (DNA Clean & Concentrator™-25 [Zymo research]) were sent for Sanger sequencing (Macrogen Europe). Obtained chromatograms were visually inspected and assembled using Sequencher 5.3 software (Gene Codes Corporation). Nucleotide polymorphisms were compared with corresponding reference sequences and reported.

Viral titration of harvested transfection supernatants was performed with a focus-forming units (FFU) assay^24,34^. Cells were seeded in 96-well plates with flat bottom and the next day, 100 μl of 10-fold serially diluted viral supernatants were added in triplicates. Cells were fixed with methanol (Sigma) after 48 h. HCV-specific immunostaining was performed with [C7-50] anti-core and mouse monoclonal 9E10 anti-NS5A antibodies, followed by addition of secondary antibody (ECL sheep anti-mouse IgG horsedish-peroxidase linked whole antibody [#NA931, GE Healthcare]) and staining with 3,3′-diaminobenzidine (DAB) substrate kit (#KBS04-500, Immunologic). The number of focus-forming units was determined with an automated cell counting system (Cellular Technology limited, CTL) and transformed to Log_10_FFU/ml.

#### Evaluation of resistance phenotype

Treatment assays with sofosbuvir consisted of dose-response assays and determination of effective 50% concentrations (EC_50_) conductedr as previously described^24,34^. Cells were plated in 96-well plates with flat bottom and the next day infected in triplicates for 24 h and thereafter treated with sofosbuvir for 48 h. Cells were then fixed and stained as described above and the counts of HCV antigen-positive cells at the different drug concentrations were related to the counts of non-treated controls. Dose-response curves were generated with the GraphPad Prism software (version 9) using the formula *Y* = Top/(1 + 10^[Log10 EC50 - *X*] * Hill slope^). One experiment with four replicates was performed for each virus. Changes in drug susceptibility for the mutant viruses were calculated as fold-EC_50_ change (EC_50_ mutant/EC_50_ original virus).

### Reporting summary

Further information on research design is available in the Nature Research Reporting Summary linked to this article.

## Supplementary information

Supplementary Information File

Reporting Summary

## Data Availability

The HCV sequence data have been deposited in GenBank database under accession codes KY620313–KY620880. The raw clinical data is available on request at http://www.stop-hcv.ox.ac.uk/data-access. Source data are provided with this paper.

## Source data

Source Data

## Code availability

The R code used to generate the results and figures from the primary analyses described above are available from the authors on request.
