# Supplementary material for: Viral genome wide association study identifies novel hepatitis C virus polymorphisms associated with sofosbuvir treatment failure
Source: Nat Commun. 2021 Oct 20;12:6105. doi: 10.1038/s41467-021-25649-6 (PMC8528821; doi:10.1038/s41467-021-25649-6)
Supplement: Supplementary file 1 — Supplementary Information File [file 41467_2021_25649_MOESM1_ESM.pdf]

Supplementary Information for the paper "Viral genome wide association study identifies novel hepatitis C virus polymorphisms associated with sofosbuvir treatment failure" by Smith et. al.

Supplementary Tables

Supplementary Table 1: Top 5 most associated amino acids with SVR in Viral GWAS in order of p-values. .A logistic regression was used to test SVR rate against each variable site in the HCV polyprotein with a count higher than 20. False discovery rate (FDR) was used to correct for multiple testing (15% FDR was used to call significant results). We only report the most associated amino acid at each site. At 15% FDR, the first three sites are significantly associated with SVR.

Position on polyprotein relative to H77	Protein	Position in Protein and Amino Acid	Available sequence data at position	SVR Rate (%)	Log odds ratio	Standard Error	P value	Q value
941	NS2	132V	506	66%	-1.27	0.29	1.05E-05	9.65E-03
1093	NS3	67V	506	77%	-1.17	0.33	4.71E-04	1.31E-01
928	NS2	119A	505	59%	-1.88	0.54	5.18E-04	1.31E-01
1944	NS4B	233V	506	58%	1.54	0.50	2.01E-03	2.95E-01
2570	NS5B	150V	501	75%	-0.76	0.27	4.92E-03	6.21E-01

Supplementary Table 2: Baseline frequency and SVR rate of amino acids at TOP sites. Only amino acids detected in more than 20 patients are included.

Protein	Position in Protein	Amino Acid	Frequency at Baseline %	SVR Rate % (n)
NS2	132	I	80.0%	86% (348/405)
		V	19.8%	66% (66/100)
NS3	67	A	53.6%	86% (232/271)
		V	43.9%	77% (171/222)
NS2	119	A	4.4%	59% (13/22)
		M	4.2%	81% (17/21)
		V	90.5%	83% (381/457)
NS5B	150	A	38.1%	88% (168/191)
		T	12.6%	83% (52/63)
		I	5%	81% (22/27)
		V	40.7%	75% (153/204)

Supplementary Table 3: Top five most associated HLA alleles with SVR and each of the TOPs. .Logistic regression was used to test for association of HLA Alleles against SVR and TOPs, a 15% FDR was used to correct for multiple testing with adjusted q values show.

HLA Allele	Log Odds Ratio	Standard Error	P Value	Q Value
SVR				
C_16	-1.12	0.43	8.24E-03	2.76E-01
A_02	0.64	0.26	1.37E-02	2.76E-01
C_05	0.92	0.38	1.42E-02	2.76E-01
B_51	-0.96	0.40	1.62E-02	2.76E-01
DRB1_01	0.77	0.37	3.89E-02	4.77E-01
NS2 119 A				
DRB1_08	1.64	0.66	1.25E-02	4.69E-01
B_51	1.48	0.62	1.61E-02	4.69E-01
A_31	1.53	0.66	2.07E-02	4.69E-01
DQA1_04	1.46	0.73	4.65E-02	7.91E-01
DQB1_04	1.36	0.72	6.07E-02	8.26E-01
NS2 132V				
B_55	1.12	0.39	4.61E-03	1.79E-01
DQB1_02	0.55	0.20	5.77E-03	1.79E-01
A_23	1.05	0.44	1.69E-02	2.33E-01
DQA1_03	-0.73	0.31	1.74E-02	2.33E-01
DRB1_04	-0.72	0.31	1.88E-02	2.33E-01
NS3 67V				
A_31	1.36	0.55	1.30E-02	4.10E-01
B_15	0.75	0.36	3.71E-02	4.19E-01
A_32	0.85	0.45	5.86E-02	4.19E-01
B_40	0.70	0.38	6.83E-02	4.19E-01
DRB5_99	-1.18	0.67	8.09E-02	4.19E-01
NS5B 150V				
DQA1_02	0.60	0.23	8.72E-03	2.96E-01
DRB1_07	0.60	0.23	8.72E-03	2.96E-01
DQB1_02	0.35	0.18	4.55E-02	6.51E-01
A_02	-0.39	0.20	5.04E-02	6.51E-01
C_12	-0.69	0.36	5.60E-02	6.51E-01

Supplementary Table 4: Primers used to generate HCV DBN3acc mutants. Position refers to the nucleotide number of the HCV genome according to the DBN3acc plasmid sequence[24].

Primer name	Position	Sequence (5’->3’)
NS2-M119V Forward	3126	CATGCTCGTGCGCTCCGTGATGGGGGGAAAATAC
NS2-M119A Forward	3126	CATGCTCGTGCGCTCCGCGATGGGGGGAAAATAC
NS2-I132V Forward	3171	CATACTGAGCGTAGGCAGGTGG
M119V/I132V Forward	3130	CTCGTGCGCTCCGTGATGGGGGGAAAATACCTCCAGATGAT CATACTGAGCGTAGGCAGG
M119A/I132V Forward	3130	CTCGTGCGCTCCGCGATGGGGGGAAAATACCTCCAGATGAT CATACTGAGCGTAGGCAGG
4136 Reverse	4139	GCCGCCACCGATGGATTC
3a27773 Forward	2778	GGCGTATGCTTGGTCGGGTG
NS3-V67A Reverse	3646	GATGTTTGGCGCCCGCGAGTG

Supplementary Figures

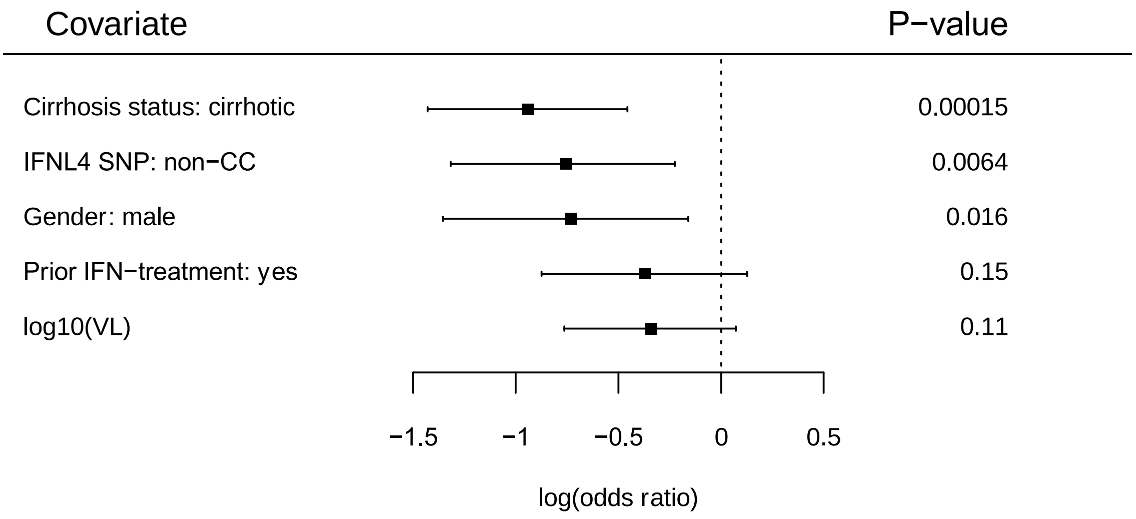

Supplementary Figure 1: Non-viral factors impact on SVR using a multivariate logistic regression. The squares show the estimated effect size (log(OR)) for each covariate and the lines show its 95% confidence interval, the p value is shown on the right. n=507 patients. For the IFNL4 SNP rs12979860 we tested non-CC genotypes (TT and CT) against the CC genotype.

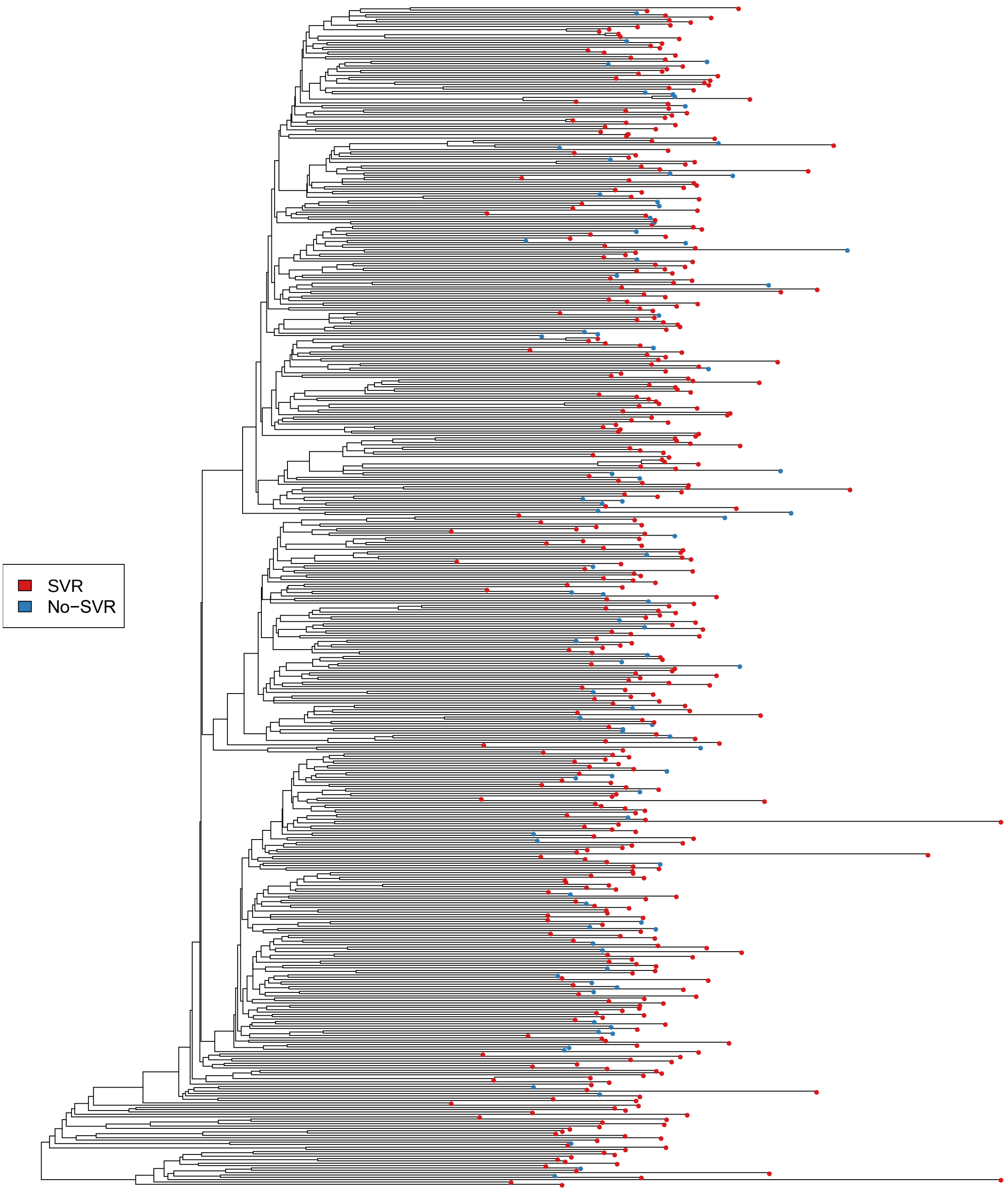

Supplementary Figure 2: Association between virus population structure and SVR. A maximum likelihood phylogenetic tree for the HCV gt3a sequences was constructed using RAXML. The colour of the tips indicates if the patient achieved SVR (red) or not (blue). The treeBreaker software did not find evidence for any clade to have a different SVR rate from the rest of the tree (Bayes factor = 0.965).

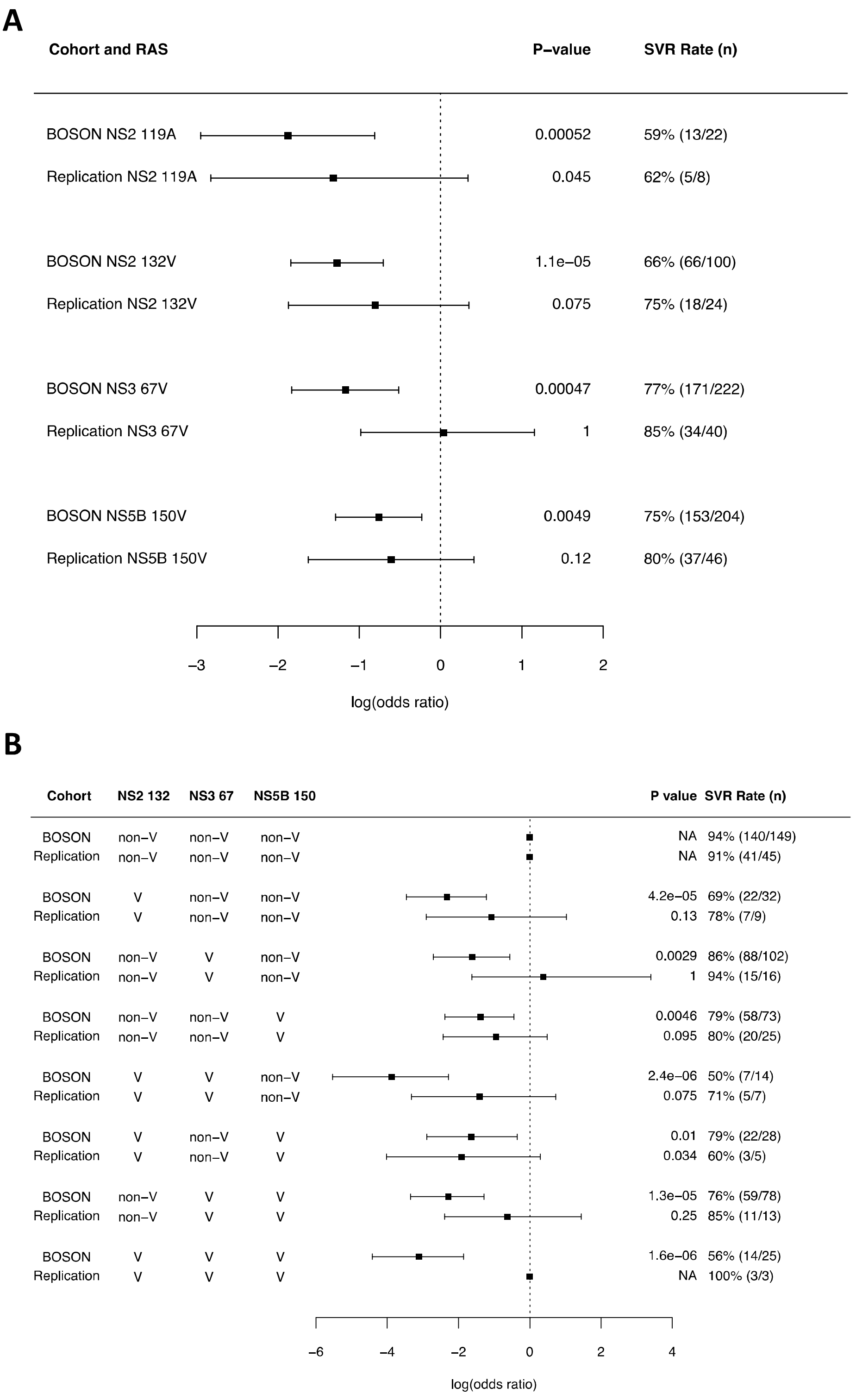

Supplementary Figure 3: A) Assocation between treatment outcome polymorphisms (TOPs) and treatment outcome in BOSON and the replication cohort. The squares show the estimated effect size (log(odds ratio)) for each TOP and the lines show its 95% confidence interval. The P values are from logistic regression. P values for the replication cohort are one-sided. **B) Covariation between the three TOP combinations and their association with**

outcome in the BOSON and replication cohorts using a multivariate logistic regression. Combinations tested are listed in the table on the left. The squares show the estimated effect size (log(odds ratio)) for each combination and the lines show its 95% confidence interval. The P values of the associations and SVR rates are shown on the right, P values for the replication cohort are one-sided.

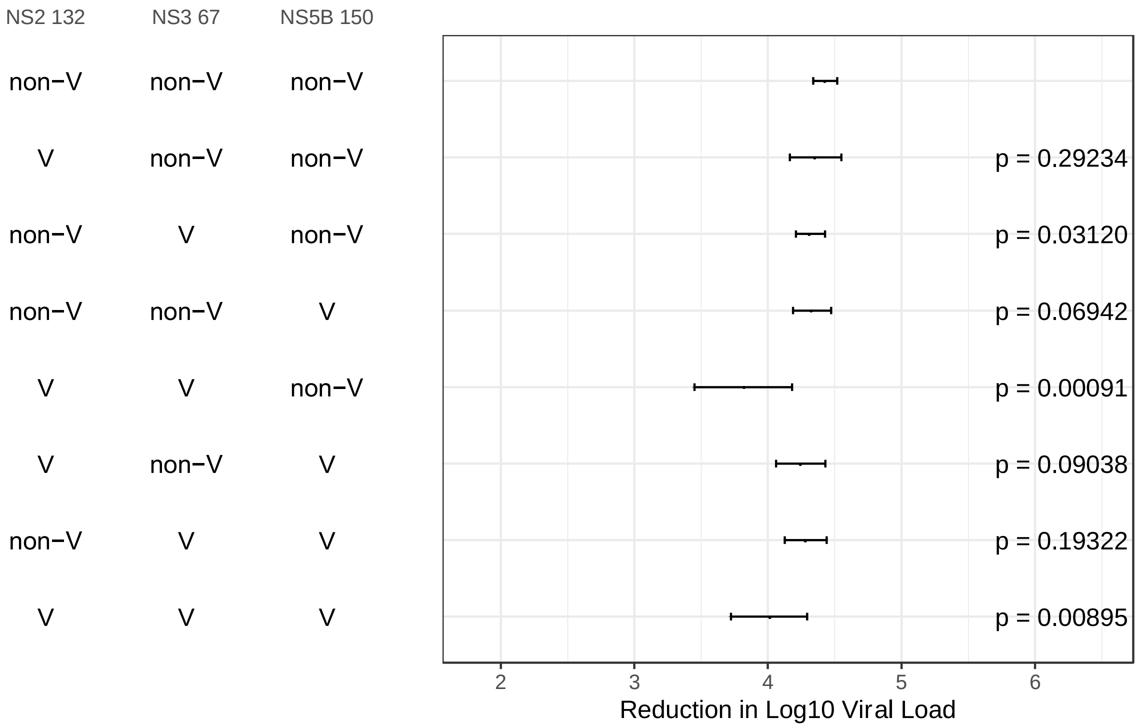

Supplementary Figure 4: Reduction in log10 viral load between baseline and week 1 of therapy for viruses carrying different combinations of TOP. n=507 patients. The mean change in viral load is shown as a black dot for each TOP and the lines indicate its 95% confidence intervals. P values for difference in mean (relative to the no TOP group) calculated using a one sided Mann-Whitney test.

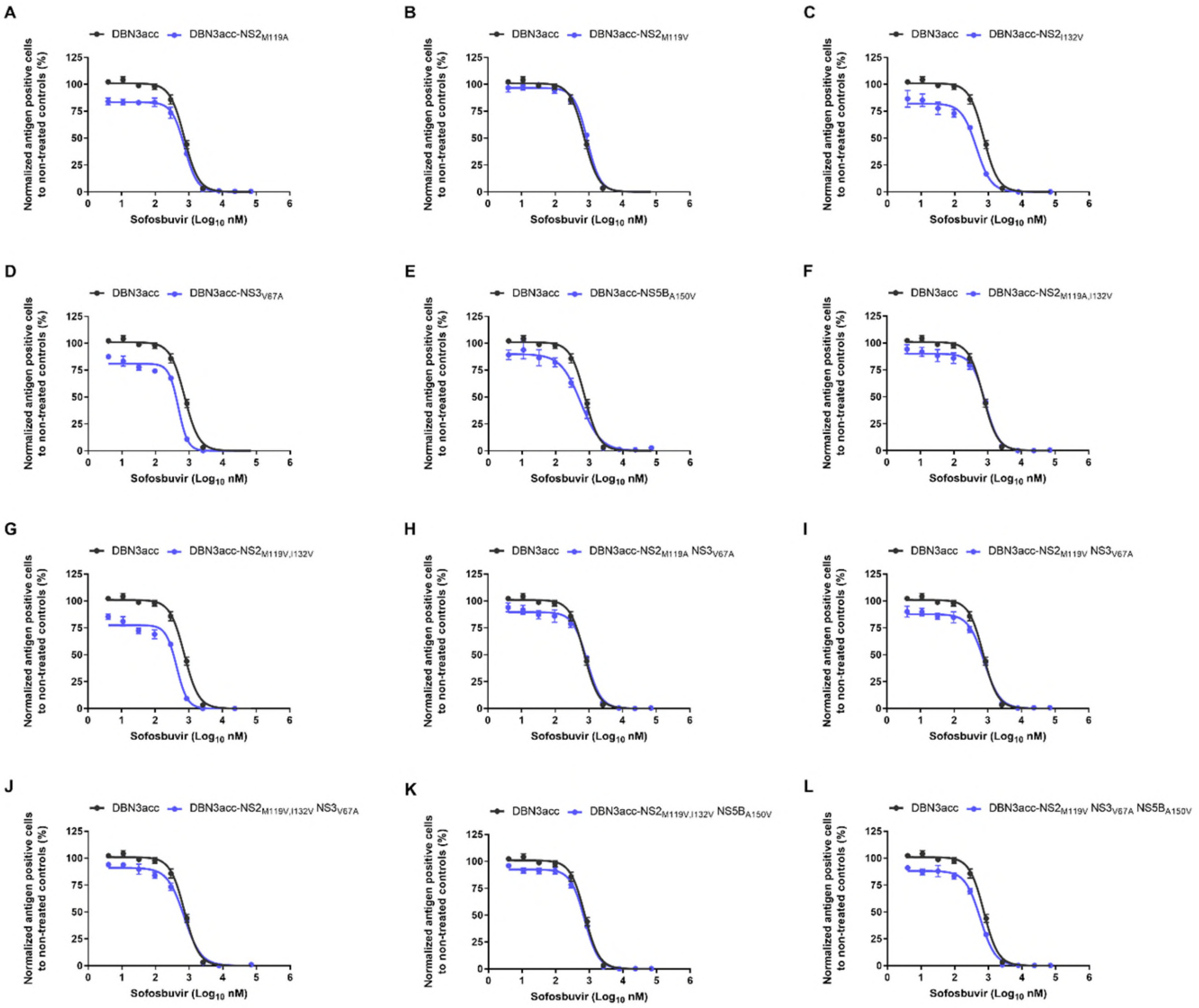

Supplementary Figure 5. Susceptibility of DBN3acc and recombinants to sofosbuvir. TOPs were engineered into the DBN3acc recombinant, and supernatant from day 10 **(A, B, D, E, F, H, I, L)**, day 12 **(J)**, day 14 **(G,K)** and day 23 **(C)** post-transfection was used to determine sofosbuvir susceptibility by drug concentration-response assays. **(A)** DBN3acc-NS2$_{M119A}$, **(B)** DBN3acc-NS2$_{M119V}$, **(C)** DBN3acc-NS2$_{I132V}$, **(D)** DBN3acc-NS3$_{V67A}$, **(E)** DBN3acc-NS5B$_{A150V}$, **(F)** DBN3acc-NS2$_{M119A,I132V}$, **(G)** DBN3acc-NS2$_{M119V,I132V}$, **(H)** DBN3acc-NS2$_{M119A}$ NS3$_{V67A}$, **(I)** DBN3acc-NS2$_{M119V}$ NS3$_{V67A}$, **(J)** DBN3acc-NS2$_{M119V,I132V}$ NS3$_{V67A}$, **(K)** DBN3acc-NS2$_{M119V,I132V}$ NS5B$_{A150V}$, or **(L)** DBN3acc-NS2$_{M119V}$ NS3$_{V67A}$ NS5B$_{A150V}$. Graphs represent sigmoidal sofosbuvir concentration-response curves for DBN3acc original (in black) and different mutants (in blue). The Y-axis indicates the HCV antigen-positive cells of treated wells normalized (percentage) to nontreated controls (mean and standard error of the mean from 4 replicates) at the different concentrations tested (X-axis, Log$_{10}$ of nanomolar sofosbuvir concentration).

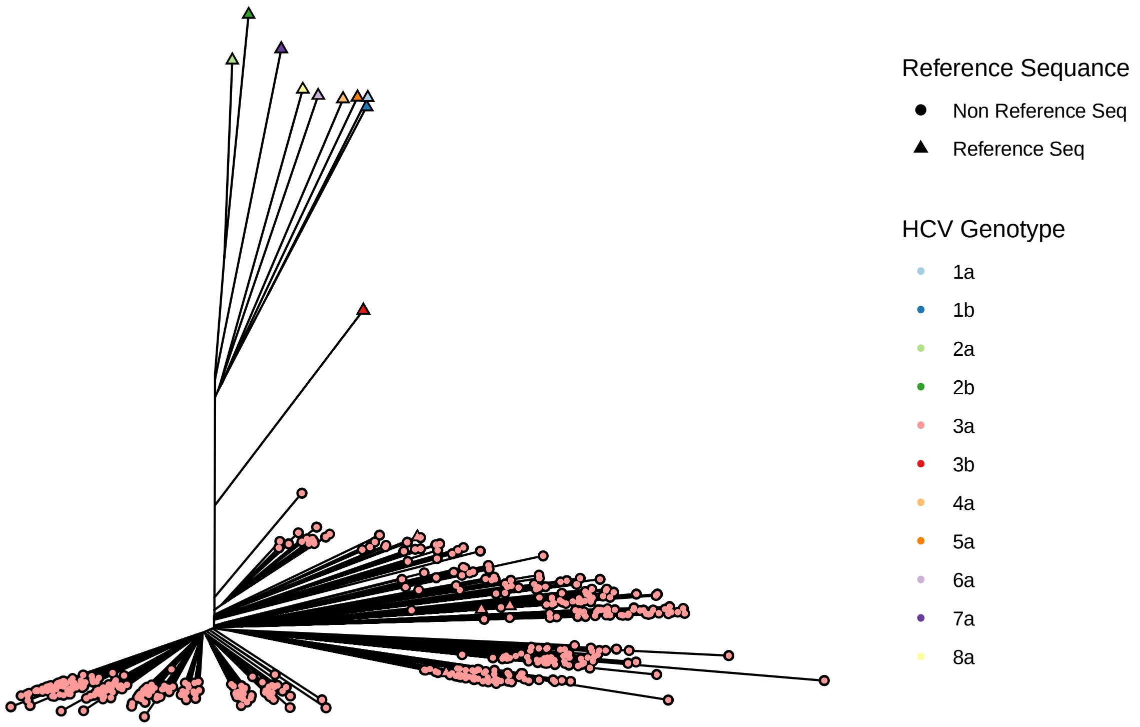

Supplementary Figure 6: Phylogenetic tree of BOSON sequences and some HCV reference sequences from International Committee on Taxonomy of Viruses.
