# Supplementary material for: Viral genome wide association study identifies novel hepatitis C virus polymorphisms associated with sofosbuvir treatment failure
Source: Nat Commun. 2021 Oct 20;12:6105. doi: 10.1038/s41467-021-25649-6 (PMC8528821; doi:10.1038/s41467-021-25649-6)
Supplement: Supplementary file 2 — Reporting Summary [file 41467_2021_25649_MOESM2_ESM.pdf]

nature research

Corresponding author(s): M. Azim Ansari

Last updated by author(s): 2021/07/26

Reporting Summary

Nature Research wishes to improve the reproducibility of the work that we publish. This form provides structure for consistency and transparency in reporting. For further information on Nature Research policies, see our Editorial Policies and the Editorial Policy Checklist.

Statistics

For all statistical analyses, confirm that the following items are present in the figure legend, table legend, main text, or Methods section.

n/a	Confirmed	
☐	☒	The exact sample size (*n*) for each experimental group/condition, given as a discrete number and unit of measurement
☐	☒	A statement on whether measurements were taken from distinct samples or whether the same sample was measured repeatedly
☐	☒	The statistical test(s) used AND whether they are one- or two-sided<br>*Only common tests should be described solely by name; describe more complex techniques in the Methods section.*
☐	☒	A description of all covariates tested
☐	☒	A description of any assumptions or corrections, such as tests of normality and adjustment for multiple comparisons
☐	☒	A full description of the statistical parameters including central tendency (e.g. means) or other basic estimates (e.g. regression coefficient) AND variation (e.g. standard deviation) or associated estimates of uncertainty (e.g. confidence intervals)
☐	☒	For null hypothesis testing, the test statistic (e.g. *F*, *t*, *r*) with confidence intervals, effect sizes, degrees of freedom and *P* value noted<br>*Give P values as exact values whenever suitable.*
☒	☐	For Bayesian analysis, information on the choice of priors and Markov chain Monte Carlo settings
☒	☐	For hierarchical and complex designs, identification of the appropriate level for tests and full reporting of outcomes
☒	☐	Estimates of effect sizes (e.g. Cohen's *d*, Pearson's *r*), indicating how they were calculated

Our web collection on statistics for biologists contains articles on many of the points above.

Software and code

Policy information about availability of computer code

Data collection	No software was used for data collection.
Data analysis	The viral seqeuncing reads were demultiplexed, low quality reads were trimmed with QUASR(v7.0120) and adapter sequences removed using Cutadapt(v1.7.1). Host derived sequences were removed using Bowtie(v2.2.4). HCV reads were selected by mapping against the 162 ICTV (International Committee on the Taxonomy of Viruses) reference sequences for mapping against the closest reference and de novo assembly (Vicuna(v1.3)), read mapping (MOSAIK(v2.2.28)), genome annotation (VFAT(v1.0)) and interpretation of variants (genewise2, Vphaser(v.2.0), Vprofiler(v1.0)). R V3.6.1 was used for statistical analysis. RAxML(v8.0) was used for estimation of phylogenies, TreeBreaker(V1.0) was used for infer the evolution of a discrete phenotype distribution on a phylogenetic tree.

For manuscripts utilizing custom algorithms or software that are central to the research but not yet described in published literature, software must be made available to editors and reviewers. We strongly encourage code deposition in a community repository (e.g. GitHub). See the Nature Research guidelines for submitting code & software for further information.

Data

Policy information about availability of data

All manuscripts must include a data availability statement. This statement should provide the following information, where applicable:

- Accession codes, unique identifiers, or web links for publicly available datasets
- A list of figures that have associated raw data
- A description of any restrictions on data availability

The HCV sequence data generated in this study have been deposited in GenBank database under accession codes KY620313 [http://www.ncbi.nlm.nih.gov/nuccore/?term=KY620313]–KY620880 [http://www.ncbi.nlm.nih.gov/nuccore/?term=KY620880]. The raw clinical data is available on request at http://www.stop-hcv.ox.ac.uk/data-access. Source data are provided with this paper.

Field-specific reporting

Please select the one below that is the best fit for your research. If you are not sure, read the appropriate sections before making your selection.

☒ Life sciences ☐ Behavioural & social sciences ☐ Ecological, evolutionary & environmental sciences

For a reference copy of the document with all sections, see nature.com/documents/nr-reporting-summary-flat.pdf

Life sciences study design

All studies must disclose on these points even when the disclosure is negative.

Sample size	568 patients from the BOSON study were included in this analysis. This includes everyone for whom we could generate HCV whole genome sequences.
Data exclusions	61 patients were excluded from this analysis as they were not infected with HCV subtype 3a. This was done to limit the impact of virus population structure on our analysis, as HCV is a highly diverse pathogen.
Replication	We attempted to replicate our viral genome wide association study in a cohort of 144 patients. Overall, the effect size estimates were similar for three of the four candidate sites, but they had larger confidence intervals due to smaller samples sizes and additional DAA's used in the therapy, this meant they did not reach statistical significance. For the in-vitro work, one experiment which included 4 replicates was performed for each virus.
Randomization	We are not performing a clinical trial study, but are analysing data that was generated as part of a clinical trial. The outcome of interest was if individuals in the study were cured of the virus or not and if any virus amino acid changes are associated with this outcome. The potential confounders were accounted for by including covariates that are potentially associated with outcome (INFL4 genotype, cirrhosis, gender, previous IFN-based treatment and log10 of baseline viral load) in our viral genome wide association study model.
Blinding	NA. There was no group allocation in our study.

Reporting for specific materials, systems and methods

We require information from authors about some types of materials, experimental systems and methods used in many studies. Here, indicate whether each material, system or method listed is relevant to your study. If you are not sure if a list item applies to your research, read the appropriate section before selecting a response.

Materials & experimental systems

n/a	Involved in the study	
☒	☐	Antibodies
☐	☒	Eukaryotic cell lines
☒	☐	Palaeontology and archaeology
☒	☐	Animals and other organisms
☐	☒	Human research participants
☐	☒	Clinical data
☒	☐	Dual use research of concern

Methods

n/a	Involved in the study	
☒	☐	ChIP-seq
☒	☐	Flow cytometry
☒	☐	MRI-based neuroimaging

Eukaryotic cell lines

Policy information about cell lines

Cell line source(s)	Huh 7.5 originally sourced from Charles Rice's lab at Rockefeller University.
Authentication	The cell line was not authenticated, however Huh7.5 and (and few derived clones) is the only cell line susceptible to HCV, thus contamination with other cell lines is unlikely.
Mycoplasma contamination	Cells were regularly tested to check that they were free of mycoplasma contamination using a commercially available kit (Universal Mycoplasma Detection Kit<br>Catalog Number 30-1012K, ATCC)
Commonly misidentified lines<br>(See ICLAC register)	No commonly misidentified cell lines were used in the study

Human research participants

Policy information about studies involving human research participants

Population characteristics	The mean age of the patients was 50, 78% of the included patients were male, 71% had cirrhosis, 86% had previous IFN-based treatment, 79% had the non-C/C IFNL4 SNP rs12979860 genotypes and the mean log10 baseline viral load of the included patients was 6.28. All these covariates were associated with outcome and included in any regression based analysis.
Recruitment	The samples used in this study came from the BOSON clinical trial which was a randomized, phase 3, open-label trial at 80 sites in the United Kingdom, Australia, the United States, Canada, and New Zealand. We did not perform a clinical trial, but used the data generated in the trial.
Ethics oversight	The clinical trial study included 80 institutions across the world as stated in the primary manuscript which describes the original clinical trial (doi: 10.1053/j.gastro.2015.07.043.). The study protocol for the BOSON clinical trail was approved by each recruiting institution's review board or ethics committee before study initiation. The study was conducted in accordance with the International Conference on Harmonization Good Clinical Practice Guidelines and the Declaration of Helsinki.

Note that full information on the approval of the study protocol must also be provided in the manuscript.

Clinical data

Policy information about clinical studies

All manuscripts should comply with the ICMJEguidelines for publication of clinical research and a completedCONSORT checklist must be included with all submissions.

Clinical trial registration	EudraCT ID: 2013-002641-11 and NCT01962441
Study protocol	https://www.clinicaltrialsregister.eu/ctr-search/trial/2013-002641-11/GB
Data collection	The data used in this study is from a randomized, phase 3, open-label trial at 80 sites in the United Kingdom, Australia, the United States, Canada, and New Zealand. Recruitment took place between October 18, 2013 and April 1, 2014.
Outcomes	The primary efficacy end point was the patient achieving a SVR12, defined as HCV RNA less than LLOQ (15 IU/mL) 12 weeks after stopping the sofosbuvir treatment drug. Plasma HCV RNA was analyzed using the Roche Cobas TaqMan HCV RNA CAP/CTM Test, v2.0 for use with the High Pure System (Roche Molecular Systems, Inc., Branchburg, NJ), which has a lower limit of quantification (LLOQ) of 15 IU/mL. Please see the relevant paper: https://doi.org/10.1053/j.gastro.2015.07.043
